# Effect of different waterlogging periods on biochemistry, growth, and chlorophyll *a* fluorescence of *Arachis hypogaea* L.

**DOI:** 10.3389/fpls.2022.1006258

**Published:** 2022-11-10

**Authors:** Shubhangani Sharma, Upma Bhatt, Jyotshana Sharma, Ahmad Darkalt, Jacek Mojski, Vineet Soni

**Affiliations:** ^1^Plant Bioenergetics and Biotechnology Laboratory, Mohanlal Sukhadia University, Udaipur, India; ^2^Department of Renewable Natural Resources & Ecology, Engineering Agricultural Faculty, Aleppo University, Aleppo, Syria; ^3^Twój Swiat Jacek Mojski, Lukow, Poland; ^4^Fundacja Zielona Infrastruktura, Lukow, Poland

**Keywords:** chlorophyll *a* fluorescence, leaf area, maximum photosystem II efficiency, oilseeds, peanut, waterlogging

## Abstract

Peanut is among the main oil crops in India with huge economic importance. The unpredictable rainy season during the growing time of peanuts causes waterlogging in peanut fields. Waterlogging triggers major environmental limitations that negatively affect the growth, physiology, and development of peanuts. Thus, the export and production of peanuts are severely affected by waterlogging. Therefore, the understanding of metabolic mechanisms under waterlogging is important to future water-stress tolerance breeding in peanuts. This study aimed to evaluate how peanuts responded to various waterlogging conditions in terms of their development, metabolic processes, and chlorophyll fluorescence characteristics. The evaluations were carried out at different stages of peanut variety DH-86 treated with waterlogging. The peanut plants were subjected to different waterlogging periods of 20, 40, 60, 80, and 100 days. The growth parameters including total dry mass, total leaf area, and total leaves number were calculated in all treatments. The phenomenological and specific energy fluxes and maximum photosystem II efficiency (F_V_/Fm) were also determined. The measurements were done statistically using PCA, G-Means clustering, and correlation analysis to explore the interaction between different physiological parameters. The waterlogging for 100 days caused a significant reduction in the total number of leaves, dry mass, and total leaf area. The most sensitive parameters are specific and phenomenological energy fluxes and Fv/Fm, which notably decreased as waterlogging duration increased. The results indicated the growth and physiological performance of the peanut cv. DH-86 was affected significantly due to waterlogging and the interaction between all these parameters in waterlogging. This research focused on how peanuts respond to waterlogging stress and provides the basis for future plant breeding efforts to improve peanut waterlogging tolerance, especially in rainy regions. This will improve the sustainability of the entire peanut industry.

## Introduction

Waterlogging stress is an important abiotic factor constraining global agricultural production, with over 12% of the worldwide agricultural land being considerably affected by waterlogging stress (Zheng et al., 2021). Waterlogging is the condition when the water content of soil reaches or approaches saturation limits (Tian et al., 2020). Over time, it slowly lowers soil oxygen levels, creates hypoxia, affects several procedures of roots like absorption of nutrients and water, root growth, and transport of water from xylem to shoot, and shifts key physiological processes including carbon metabolism, growth, production, and gas exchange processes (Toral-Juárez et al., 2021). These are the results of early stomatal closure, which is driven by declines in root hydraulic conductance and concentration of ABA, which restrict photosynthetic rates, RuBisCo (ribulose-1,5-bisphosphate carboxylase/oxygenase) carboxylation rate, and water loss in plants (Loreti et al., 2016; Yamauchi et al., 2018).

Under continuous waterlogging, the photosynthetic performance of plants is reduced at the biochemical and photochemical levels, and primarily reactive oxygen species (ROS) production causes the flow of electron transfer in the photosystems to be interrupted, increasing the lipid peroxidation processes. This increases the degradation of photosynthetic pigments and causes an excessive accumulation of energy activation at the photosystem level, which can cause a more drastic reduction in the photosystem's ability to produce oxygen. (Loreti et al., 2016; Toral-Juárez et al., 2021). Waterlogging also impacts metabolic activities like plant photosynthesis. While waterlogging-sensitive plants experienced a sudden decline in photosynthetic rate, waterlogging-tolerant plants did not see as much of a reduction (Zeng et al., 2020). Additionally, the deficiency of oxygen caused by waterlogging stress triggered anaerobic respiration to supply the cell energy (Da-Silva and do Amarante, 2020). Lactic acid and ethanolic fermentation are typically two of the fermentation pathways involved in anaerobic respiration. Therefore, as indications, the enzyme activity in these fermentation pathways, such as lactate dehydrogenase (LDH) and alcohol dehydrogenase (ADH), are changed. For example, ADH and LDH will be activated to produce ATP for plants, increasing their capacity to handle the stress of waterlogging (Dennis et al., 2000; Xu et al., 2016).

During waterlogging, the expression of several genes associated with waterlogging shifted. For instance, the expression of *ACC synthase 6* (*ACS6*), a gene involved in ethylene synthesis has changed in roots and leaves. However, the expression of *ACS6* varied with the tissues of the plant being upregulated in the leaves and downregulated in the roots. Eight ethylene response factors (ERFs) were affected by waterlogging in distinct manners by the plant tissues. After waterlogging, the expression levels of numerous genes involved in cell-wall remodeling were also found in root and leaf tissues. Under waterlogging treatment, the expression of several genes was linked to aerenchyma formation (*GhXTH, GhXTH1*, and *GhXTH3*), and cell expansion (*GhEXPA2*) increased in the roots but decreased in the leaves. Waterlogging stress in cotton leaves reduced the expression of Chlorophyll a/b-binding (*GhLHCB*), a gene involved in the light-harvesting complex of photosystem II (PSII). In the leaves of waterlogged plants, two NO synthesis-related genes (*GhNOS1* and *GhNOS2*) were significantly down-regulated. The antioxidant gene superoxide dismutase (*GhCSD*) was up-regulated in cotton leaves. Under waterlogging, the important low oxygen-induced genes, *GhADH* and *GhPDC*, were upregulated in both root and leaves (Zhang Y. et al., 2015).

The balance of several hormones is the basis for ensuring that plants experience appropriate physiological metabolism, growth, and development. Endogenous plant hormones are closely involved in the control of the entire life process of plants (Miransari and Smith, 2014; Wang et al., 2020). By complex signaling, the plant modulates the balance between the synthesis and transport of plant hormones and controls the response to waterlogging. In the mechanism of waterlogging tolerance, plant hormones serve as important endogenous signals (Yamauchi et al., 2020). An increase in the content of signaling agents (SA) may have a significant role in waterlogging stress tolerance. As a signaling agent, salicylic acid can cause changes in the physiological traits of waterlogged plants. Spraying exogenous SA of waterlogging can significantly increase the activity of ethanol dehydrogenase, protective enzymes like POD and CAT, and the content of proline in leaves and roots, preventing damage to leaves and root membranes and stabilizing leaves' and roots' photosynthetic capacity.

Global consumption of oilseeds and vegetable oils obtained from oil-yielding crops is increasing, especially in emerging nations where the population is booming. Dietary preferences are changing and the living standards and purchasing power of consumers are continuing to rise (Zafar et al., 2019; Xu et al., 2021). Although organic peanut oil is becoming more popular, conventional peanut oil still dominates the market with a share of over 97%. Global peanut oil sales were between 3.5 and 4.5% of the value of the 2020 worldwide market for vegetable oil. The global peanut oil market experienced a value of 3.4% from the year 2016 to 2020. Due to growing consumer awareness of its health benefits, the industry of food processing is the main factor fueling the market expansion for peanut vegetable oil. This demand has compelled important groundnut oil producers to create high-quality oil that lives up to consumers' expectations. As a multipurpose oil-seed legume, peanuts have several advantages. In addition to improving soil quality, peanut seeds have substantial economic, medical, and nutritional benefits. The active substances phenolics, flavonoids, polyphenols, and resveratrol are abundant in peanuts. Furthermore, the contribution of peanuts to biological nitrogen-fixing is very important.

Recent studies on how peanuts react to abiotic challenges such as drought, salt, heat, and waterlogging stress have been considered. In particular, the necessity of growing and consuming peanuts for human use in light of changing global climate is paramount to ensure food security (Akram et al., 2018). Previous research has shown that the photosynthetic system of waterlogged peanut leaves was disrupted, which limits its ability to absorb CO_2_ and decreased overall photosynthetic efficiency. The pod-filling stage was the most susceptible stage to waterlogging (Mitchell et al., 2013; Miransari and Smith, 2014; Ahmad et al., 2021; Stasnik et al., 2022). Waterlogging at this time drastically reduced the number and weight of pods per plant, which ultimately resulted in a decline in peanut productivity (Bishnoi and Krishnamoorthy, 1992; Zeng et al., 2020). Due to global warming, waterlogging constitutes an increased risk for peanut growth and development (Schiermeier, 2011). On the other hand, little is known about how peanuts react to waterlogged soil. Plants can respond in several ways depending on the severity and length of the stress. Thus, it is essential to understand the physiological changes brought on by increases in the soil water (Liu et al., 2021; Zeng et al., 2022).

This study aimed to evaluate how peanut plants respond to water availability in terms of growth and photochemical efficiency. The effects of changes in chlorophyll “*a*” fluorescence and leaf water potentials on growth indices including total leaf number, leaf area, total dry mass, and physiological functioning were investigated. This knowledge is essential to understanding the responses of peanut plants to waterlogging and the negative consequences of waterlogging on the physiological function of *Arachis hypogaea* plants, which are widely sown in Rajasthan's dry region. The significance of this research will help the expansion of the food industry which is the fastest-growing industry worldwide, especially in developing countries (Arduini et al., 2016; Masoni et al., 2016; Chen et al., 2020; Cotrozzi et al., 2021). Manufacturers are adopting and developing novel processing technologies in response to the growing demand for nutritious food and food with functional properties. Processed and convenience food sales have increased as a result of rising disposable income brought on by economic growth, especially in developing nations (Pampana et al., 2016). The market for processed foods is estimated to expand due to an increase in population and consumers' busy lifestyles. Peanut oil harbors many healthy and nutritional advantages, and the increase in the world's demand for edible oils is an excellent sign for the industry.

## Materials and methods

### Plant material collection, growth conditions, and waterlogging treatments

**Figure 1:**
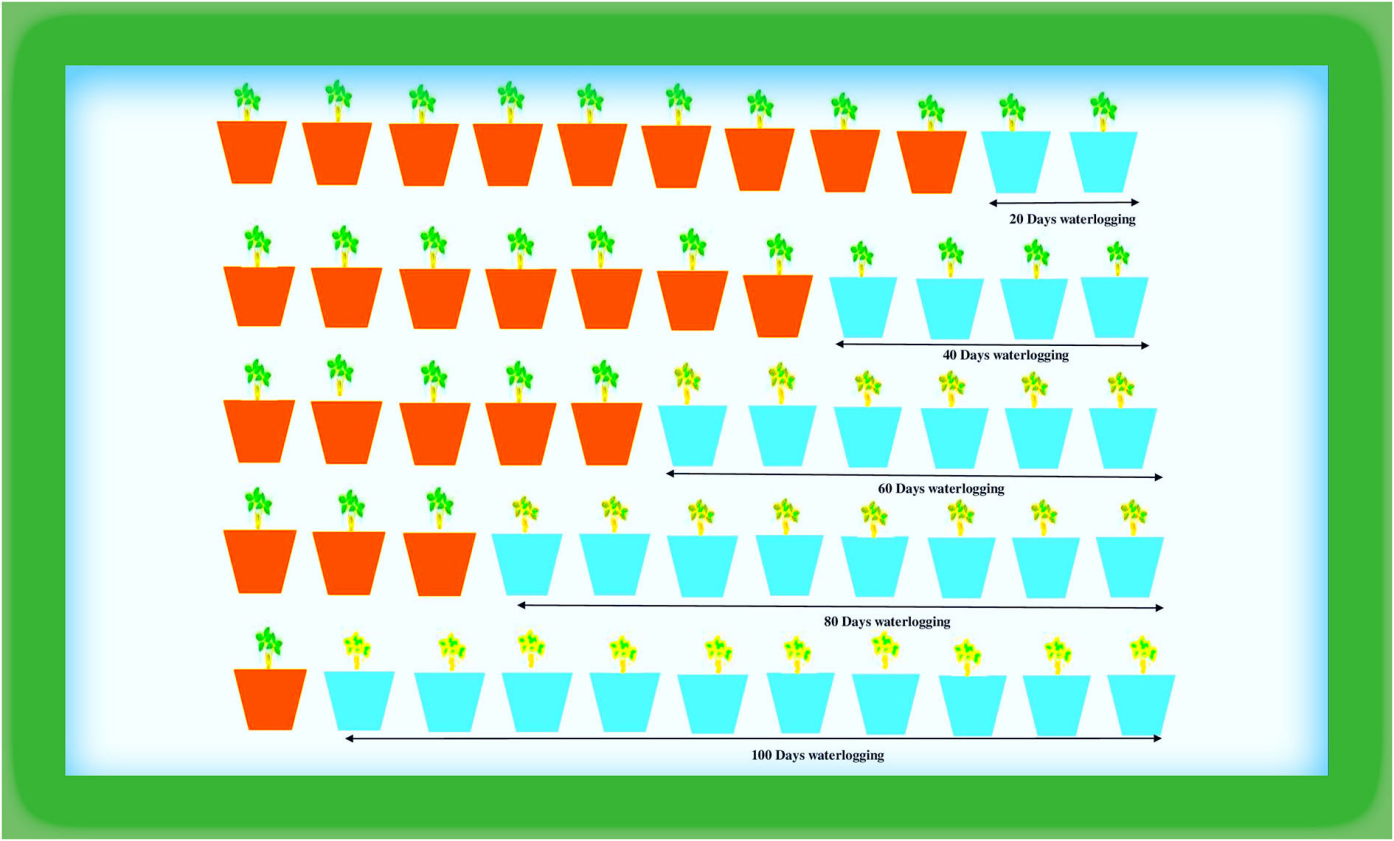
Diagrammatic representation of the experiment.

The plant material (DH-86) was collected from MPUAT, Udaipur [24°58′N, 73°71′E], and pot experiments were carried out under semi-controlled conditions in a greenhouse (Figure 1). The greenhouse experienced typical climatic conditions during the study, including 31.04°C average climatic conditions and 67.93% relative humidity. The seedlings were put in pots with sandy loam soil that was 28 cm wide by 22 cm long (05 days after seed sowing).

**Figure 2:**
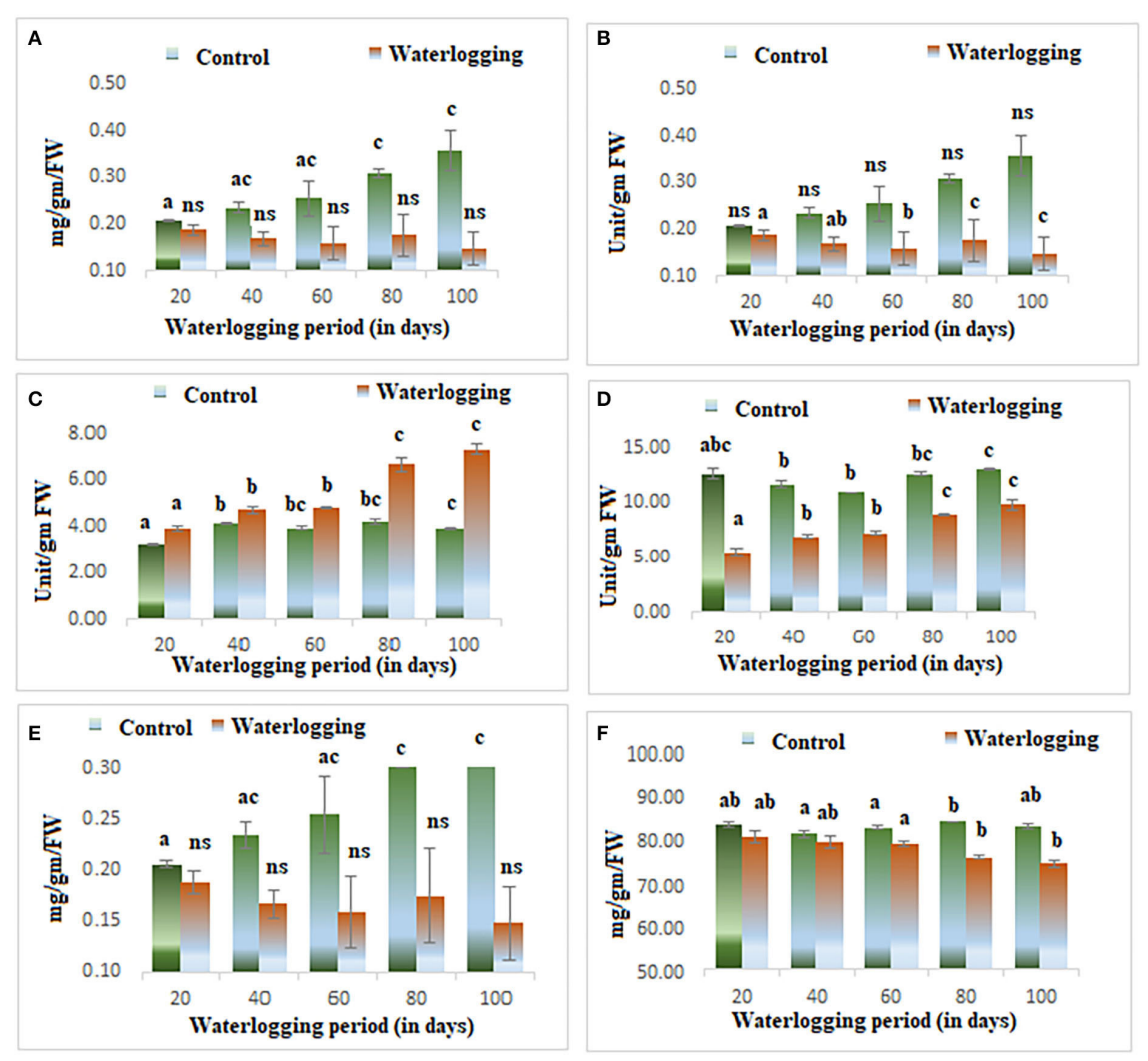
Biochemical changes in peanuts under different waterlogging treatments **(A)** Chlorophyll content **(B)** Activity of superoxide dismutase **(C)** Catalase activity **(D)** GPOD activity **(E)** Soluble sugar content **(F)** Starch content. The letters are the result of post hoc test.

The peanut plants underwent the following waterlogging treatments in August 2021. To sustain moisture levels at the field capacity of the soil, plants with a better supply of water were maintained as controls. In the first treatment (T1), plants were waterlogged for 20 days; in T2 for 40, in T3 for 60, in T4 for 80, and T5 for 100 days. The treatments included sustaining a daily layer of water in the pots that was 3–5 cm over the ground. The treatment was applied daily for 100 days (Figure 2). The purpose of this process was to maintain the same timing and climatic conditions for the plants. All measurements were taken at 100 days following the waterlogging-treatment application. Random biological replicates were taken for the analyses of data as per Blainey et al. (2014) and Vinson et al. (2018).

### Measurements of growth parameters

The total leaf area (TLA), the total number of leaves (TNL), and the plants' dry mass (DM) were all measured. The area of the leaf was measured by quantifying the width and length of each fully expanded leaf and applying the given formula, as suggested by Unigarro-Muñoz et al. (2015). The TNL was determined by direct counts on the plants.


$$\begin{array}{l} EAF\;=\;0.99927\;\times \;(Length\;\times \;(-0.14757\;+\;0.60986\;\times \\ \;Width) \end{array}$$

where:

EAF, estimate of leaf area;

length, leaf length;

width, leaf width.

The roots and stems of plants were taken individually and put in labeled paper bags for the dry mass calculation. The material was dried for 72 h in the oven set to 65°C. The leaf dry mass, stem dry mass, root dry mass, and total dry mass of the plants were then determined using a 0.01 g precision balance.

### Biochemical measurements

#### Soluble sugars

About 0.5 g of the sample was extracted with 80% ethanol before being used to test the water-soluble sugars using the anthrone method. The extract was centrifuged for 30 min. The freshly made anthrone reagent was combined with the obtained supernatant. For 20 min, samples were incubated at 80°C. Utilizing a spectrophotometer, absorbance at 620 nm was measured after cooling (Arthur Thomas, 1977).

#### Starch content

A method based on acid hydrolysis described by McCready et al. (1950) was used to estimate starch.

#### Antioxidative enzyme activity

By evaluating the SOD's ability to prevent the photochemical reduction of nitro blue tetrazolium (NBT) at 560 nm, the SOD activity was evaluated using a spectrophotometer. About 100 ml of L-methionine, 100 ml of NBT, 10 ml of riboflavin, 100 ml of enzyme extract, and 2.7 ml of Na_2_CO_3_ were added to the reaction mixture (0.05 M). After being exposed to white fluorescent light for 10 min, the process was terminated by leaving the tubes in the dark for 8 min, and at 560 nm, absorbance measurements were calculated. The amount of SOD enzyme needed to achieve a 50% inhibition of the NBT reduction was defined as one unit of the SOD enzyme activity (Kumar et al., 2021).

The impact of variations in absorbance at 436 nm was determined for periods ranging from 15 s to 5.0 min and was used to calculate the GPOD activity. The reaction was started by adding H_2_O_2_ to the reaction mixture, which also included 1.0 ml of guaiacol (1.0 %) and 1.7 ml of phosphate buffer (0.05 M, pH 7.0). Unit enzyme activity was used to express the amount of enzyme required to convert the substrate in 1 min (Soni et al., 2021).

The CAT activity was determined by monitoring H_2_O_2_ consumption at 240 nm. About 120 ml of enzyme extract, 80 ml of 500 mM H_2_O_2_, and 2.8 ml of potassium phosphate buffer were used in the reaction mixture (50 mM). The quantity of enzyme required to reduce 1.0 mM of H_2_O_2_ per minute was used to define one unit of catalase activity (Singh et al., 2021).

#### Chlorophyll content measurement

The fresh leaves (300 mg) of all treated plants were collected. The leaves were cut into small pieces, put in a mortar with liquid nitrogen, and then ground with a pestle. The powder was transferred to a 15 ml Falcon tube, and 5.0 ml of 80% acetone was added and stirred for 15–30 min in the dark (because chlorophylls degrade under light conditions). The supernatant was transferred to a fresh centrifuge tube and stored in the dark after centrifuging the tube at 4°C for 15 min at 3,000 rpm. Steps three and four were replicated and the supernatant was added to the centrifuge tube. Spectrophotometry was used to completely combine the contents in the tube and 80% acetone was used as a blank control to determine the absorbance of chlorophyll content. The levels of chlorophyll were measured using the following formula:


$$\begin{array}{l} Chlorophyll\;a\;(in\;mg/g)\;=\;[12.7\;\times \;A_{663}\;-\;2.69\;\times \;A_{645}]\\ \;\times \;V/1,000\times W \end{array}$$

$$\begin{array}{l} Chlorophyll\;b\;(in\;mg/g)\;=\;[22.9\;\times \;A_{645}\;-\;4.86\;\times \;A_{663}]\\ \;\times V/1,000\times W \end{array}$$

$$\begin{array}{l} Chlorophyll\;a+b\;(in\;mg/g)\;=\;[8.02\times A_{663}\;+20.20\;\times \\ \;A_{645}]\;\times V/1,000\times W \end{array}$$

where *V* = volume of the extract (ml); *W* = weight of fresh leaves (g) (Arnon, 1949).

### Physiological measurements

#### Chlorophyll “a” fluorescence parameters

After 100 days, all samples were dark-adapted for 1 h before the measurements. OJIP transient for fresh leaves and all measurements were taken by using a plant efficiency analyzer, Handy PEA (Hansatech Instruments, Kings Lynn, and Norfolk, U.K.). All samples (control and 20, 40, 60, 80, and 100 days of waterlogged plants) were analyzed using light of 650 nm with a light intensity of 3,000 μm photons m^−2^s^−1^ emitted by three LEDs (light-emitting diodes). The Fo and Fm were measured at 720 nm. Fo was measured at 50 μs. Other different biophysical parameters were measured by using the formula in Table 1.

**Table 1:** Chlorophyll fluorescence parameters were analyzed in the present study.

**S.no**.	**Parameters**	**Formula**
1.	Fo	Initial reliable fluorescence measured at 50 μs
2.	F_I_	Fluorescence at 2 ms (J-level)
3.	F_J_	Fluorescence at 30 ms (I-level)
4.	Fm	Maximum fluorescence at saturating illumination (peak of OJIP)
5.	RC/CS	(ABS/CS) (RC/ABS)
6.	ABS/CS	Approximately proportional to F_O_
7.	TR/CS	(ABS/CS) (TR/ABS)
8.	ET/CS	(ET/RC) (RC/CS)
9.	DI/CS	(ABS/CS) − (TR/CS)
10.	Fv/Fo	F_M_ − F_50μ*s*_/F_50μ*s*_
11.	ABS/RC	[(TR/RC)/(TR/ABS)]
s12.	TR/RC	(M_O_/Vj)
13.	ET/RC	Mo·(1/Vj)·(1−Vj)
14.	DI/RC	[(ABS/RC) − (TR/RC)]
15.	Vj	Variable fluorescence − Fj − Fo
16.	φ P_o_	1 − (Fo/F_m_)
17.	φ E_o_	[1 − (Fo/F_m_)] −Ψ_O_
18.	φ D_o_	1 −φPo − (Fo/ F_M_)
19.	Ψ_O_	ET/TR = (1 − V_J_)
20.	Ψ_O_/(1 −Ψ_O_)	1 − Vj/[1 − (1 − Vj)]
21.	φ_o_/(1 −φ_o_)	Conformation term for primary photochemistry
22.	PIcsm	RC/CS × [ φpo/(1 −φpo)] × [Ψo/(1 −Ψo)]
23.	PIabs	(RC/ABS) × [φ _Po_/(1 −φ _Po_)] × [Ψ _O_/(1 −Ψ _O_)

### Data analysis

For the data analysis, preparation of graphs, and measuring average or standard error, MS Excel was used. The ANOVA and *post-hoc* analyses were performed in IBM SPSS Statistics 28.0.0.0. All data in this study is the average of five biological replicates selected randomly from all control and treated pots. The G-Means clustering, principal component analysis, and correlation analysis were done using XLSTAT 2020.

## Results

### Changes in growth parameters of the plants

Waterlogging seriously hampered the growth rate and productivity of plants. In this study, the growth rate was measured as the total number of leaves, total leaf area, and dry mass of the peanut plants. All these parameters were significantly altered in all waterlogging treatments. The waterlogging condition declined the total number of leaves, total leaf area, and dry mass of peanut plants. The highest reduction was seen in 100 days of waterlogged peanut plants as compared to other waterlogging-treated plants (Table 2).

**Table 2:** The results of growth parameters under different waterlogging conditions.

**S.no**.	**Waterlogging treatment**	**Total number of leaflets (TNL)**	**Total leaf area**	**TDM of plants**
1.	20 days	12 ± 11	15 ± 1	22.43 ± 2
2.	40 days	10 ± 1	15 ± 1	18.67 ± 2
3.	60 days	07 ± 1	12 ± 1	16.69 ± 2
4.	80 days	06 ± 1	10 ± 1	12.47 ± 2
5.	100 days	03 ± 1	07 ± 1	9.43 ± 2

### Changes in chlorophyll content

Wilting and senescence of leaves were observed in all treated plants. The level of chlorophyll decreased in all waterlogged plants. The decline in chlorophyll content was the most obvious sign of waterlogging, and it is the first indicator of damage due to waterlogging. The chlorophyll content reduced significantly from 20 to 100 days of waterlogged plants and the highest reduction was seen in 100 days of waterlogged plants. However, the plants were completely dead in 100 days of waterlogging conditions (Figure 2A).

### Changes in antioxidant enzymes activity

Higher antioxidant enzyme activity was reported in all treated plants. The activity of catalase and superoxide dismutase remained constant in 20, 40, and 60 days of waterlogged plants, after that it increased. The activity of both enzymes was maximum in 100 days of waterlogged plants. The activity of GPOD was higher throughout the experiments, and it gradually increased as the waterlogging duration increased (Figures 2B–D).

### Changes in starch and soluble sugar content

The productivity of the plants was measured by the presence of soluble sugar and starch content. The starch and soluble sugar were significantly reduced in all treated plants (20, 40, 60, 80, and 100 days of waterlogged plants). The starch content was reduced due to the lower assimilation of photoassimilates. The decrease in photoassimilation is associated with the lower efficiency of photosynthesis. The efficiency of photosynthesis was determined using chlorophyll fluorescence analyses, and all parameters of the photochemistry of PSII were modulated in this study (Figures 2E,F).

### Changes in chlorophyll fluorescence parameters

#### Effect of waterlogging stress on fluorescence

**Figure 3:**
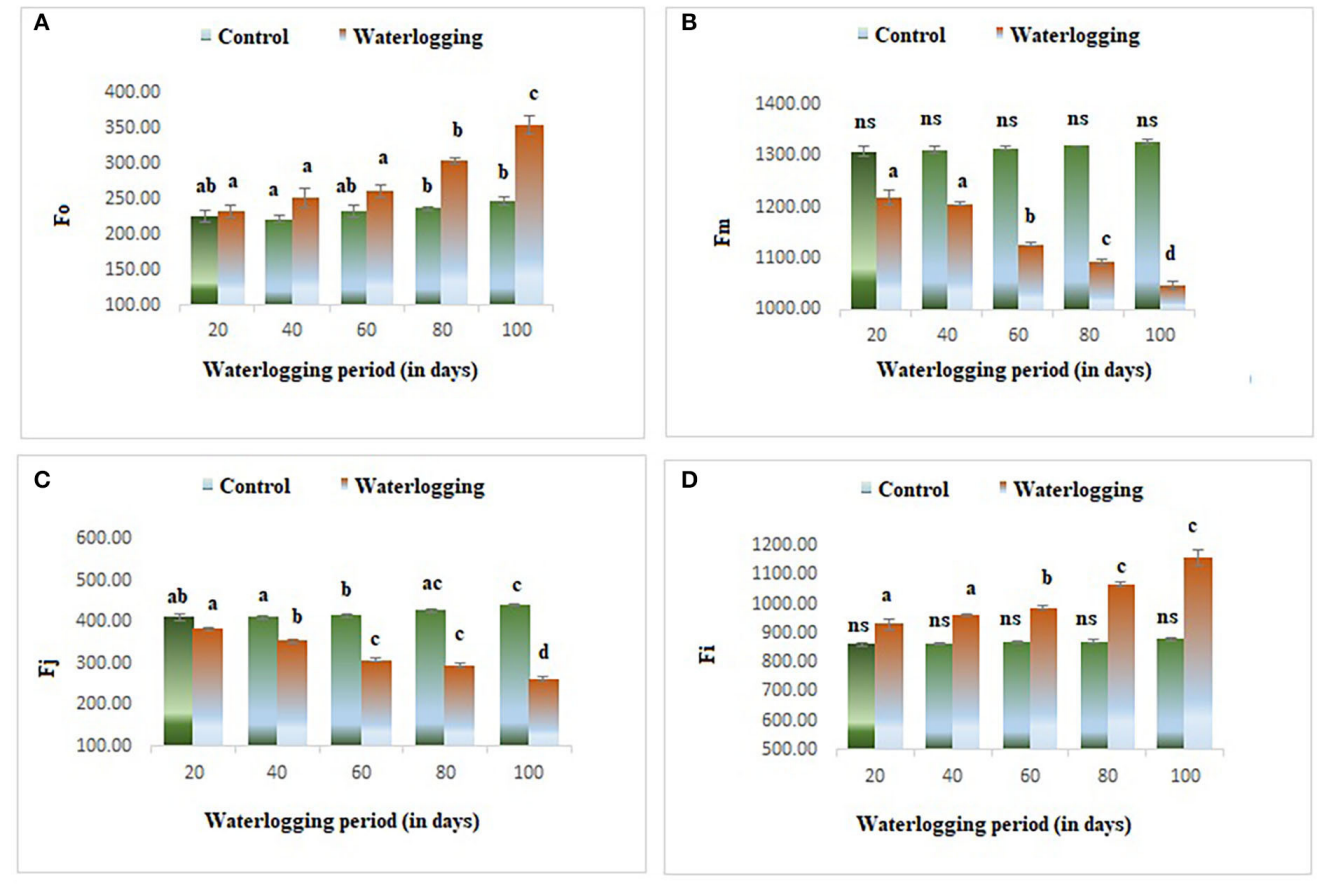
Waterlogging-induced changes in different fluorescence parameters **(A)** Fo **(B)** Fm **(C)** Fj **(D)** Fi. The letters are the result of post hoc test.

**Figure 4:**
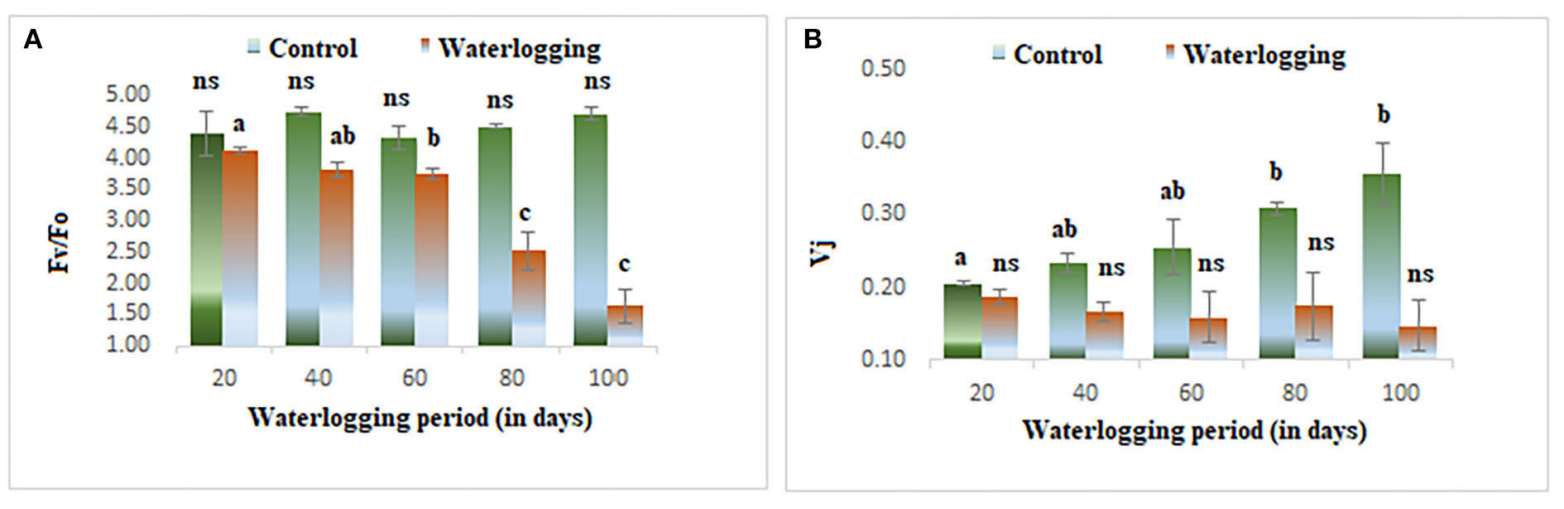
Waterlogging-induced changes in **(A)** Fv/Fo **(B)** Vj. The letters are the result of post hoc test.

The value of minimal fluorescence (Fo) increased gradually in waterlogged plants as compared to the control. The maximum value of Fo was found in 100 days waterlogged peanut plant. In the control, the value of Fo remained constant throughout the experiment (Figure 3A). The maximal fluorescence (Fm) declined as the waterlogging duration increased. The Fm remained the same in 40 days of waterlogged plants and then decreased gradually and in 100 days of waterlogged plants, the Fm value reached the lowest (Figure 3B). The fluorescence at *j* and *i* level (Fj) and (Fi) were also altered in peanuts under waterlogging. The Fj was reduced in plants under waterlogging conditions of 60, 80, and 100 days. On the other hand, the Fi was increased dramatically in all treated plants, and the maximum value was found in 100 days of waterlogging treated plants (Figures 3C,D). The variable to minimum fluorescence ratio (Fv/Fo) was reduced as the waterlogging duration increased in our experiments. The Fv/Fo remained the same in 20, 40, and 60 days of waterlogged plants and then declined drastically in 80 and 100 days of waterlogged plants. The Vj was decreased in all treated plants and the minimum value was reported in 100 days of waterlogged plants (Figures 4A,B).

#### Effect of waterlogging on phenomenological energy fluxes

**Figure 5:**
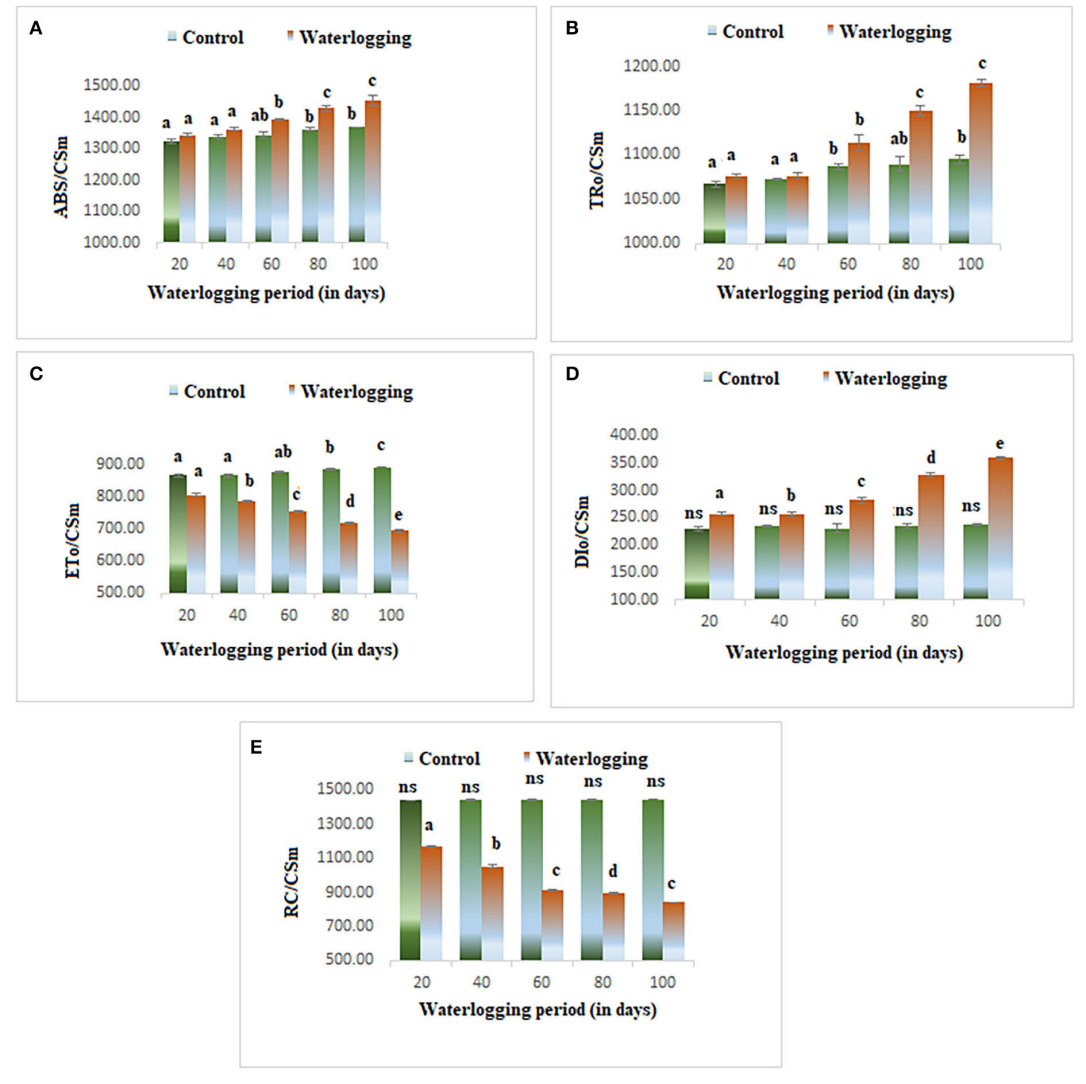
Waterlogging-induced changes in phenomenological energy fluxes **(A)** ABS/CSm **(B)** TR/CSm **(C)** ET/CSm **(D)** DI/CSm **(E)** RC/CSm. The letters are the result of post hoc test.

**Figure 6:**
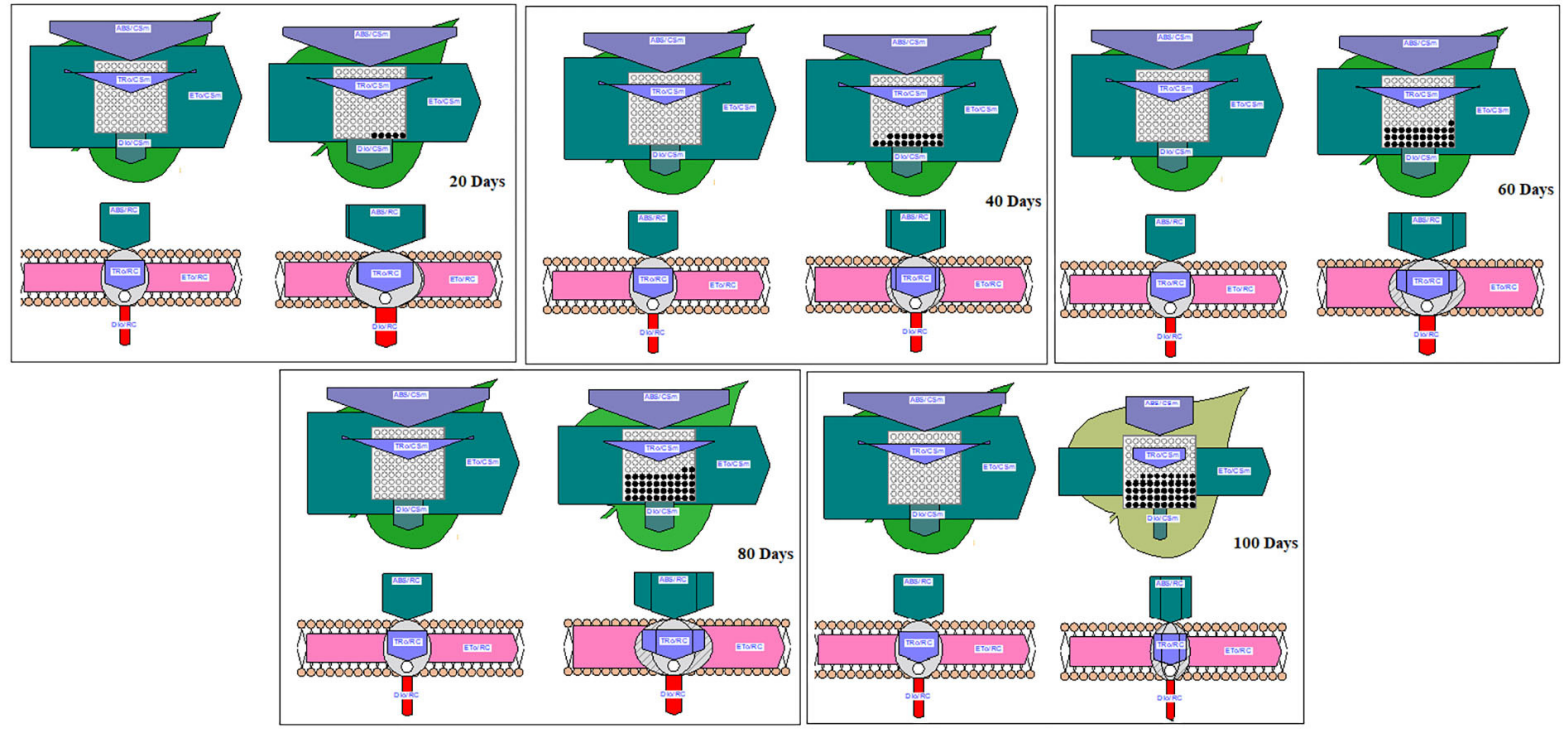
Pipeline and membrane models of peanut chlorophyll fluorescence under different waterlogging treatments.

Phenomenological energy fluxes (ABS/CSm, TR/CSm, and DI/CSm) were enhanced in all treated plants as compared to the control; however, ET/CSm and RC/CSm dropped significantly in all treated plants (Figure 5). The phenomenological energy fluxes are represented as an energy pipeline leaf model in Figure 6.

#### Effect of waterlogging on specific energy fluxes

**Figure 7:**
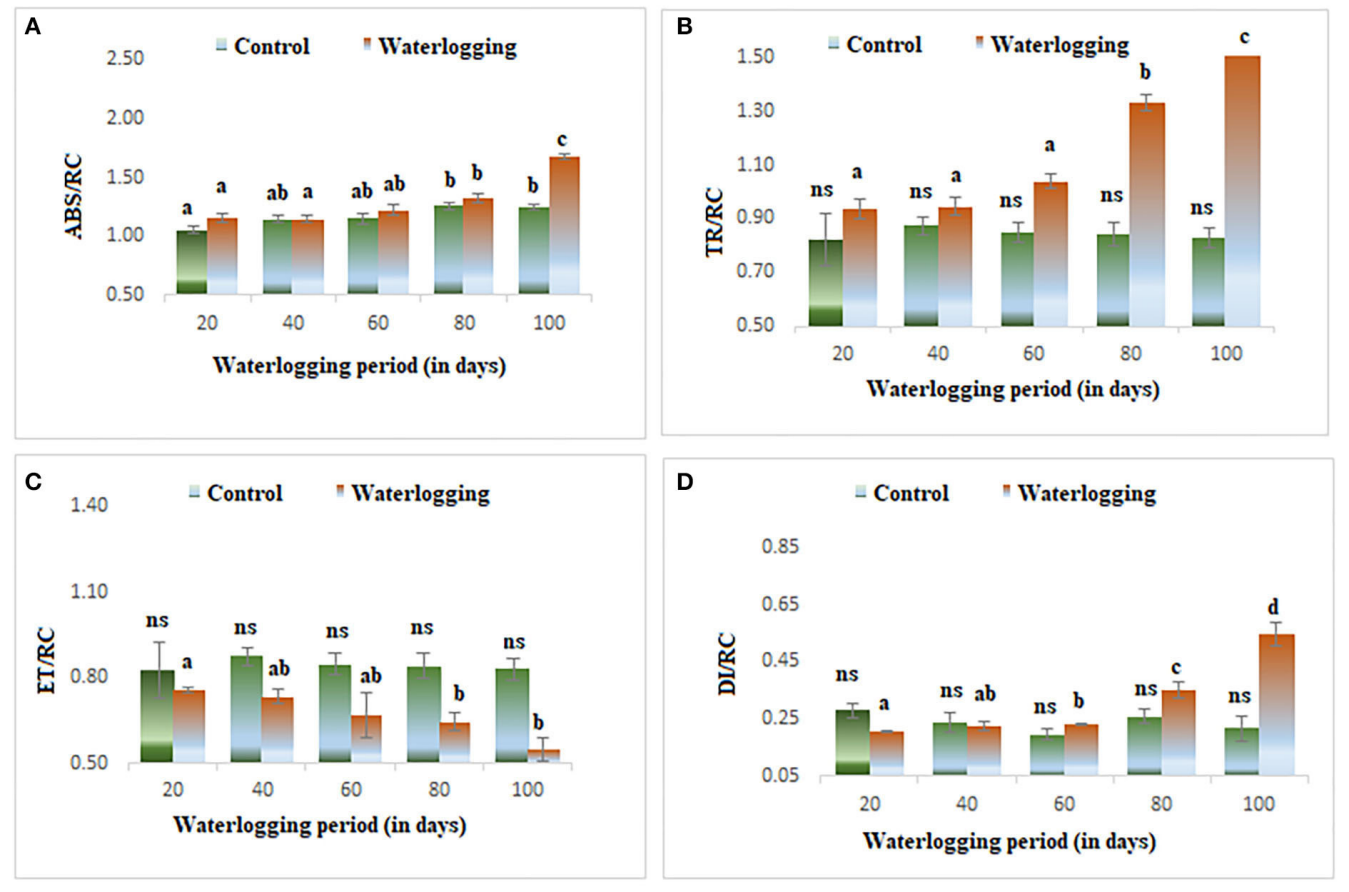
Waterlogging-induced changes in specific energy fluxes **(A)** ABS/RC **(B)** TR/RC **(C)** ET/RC **(D)** DI/RC. The letters are the result of post hoc test.

There was a rise in specific energy fluxes (ABS/RC, TR/RC, and DI/RC) in all treated plants and a higher value was found in 100 days of waterlogged plants of peanut. However, the ET/RC was declined in waterlogged condition. The lowest value of ET/RC was found in 100 days of waterlogged plants (Figure 7). The specific energy fluxes are represented as the thylakoid membrane model in Figure 6.

#### Effect of waterlogging on performance indices

**Figure 8:**
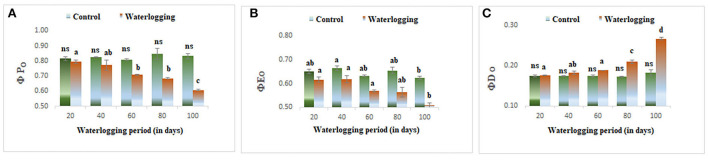
Waterlogging-induced alteration in **(A)** quantum yield of photosynthesis **(B)** quantum yield of electron transport **(C)** quantum yield of dissipation. The letters are the result of post hoc test.

**Figure 9:**
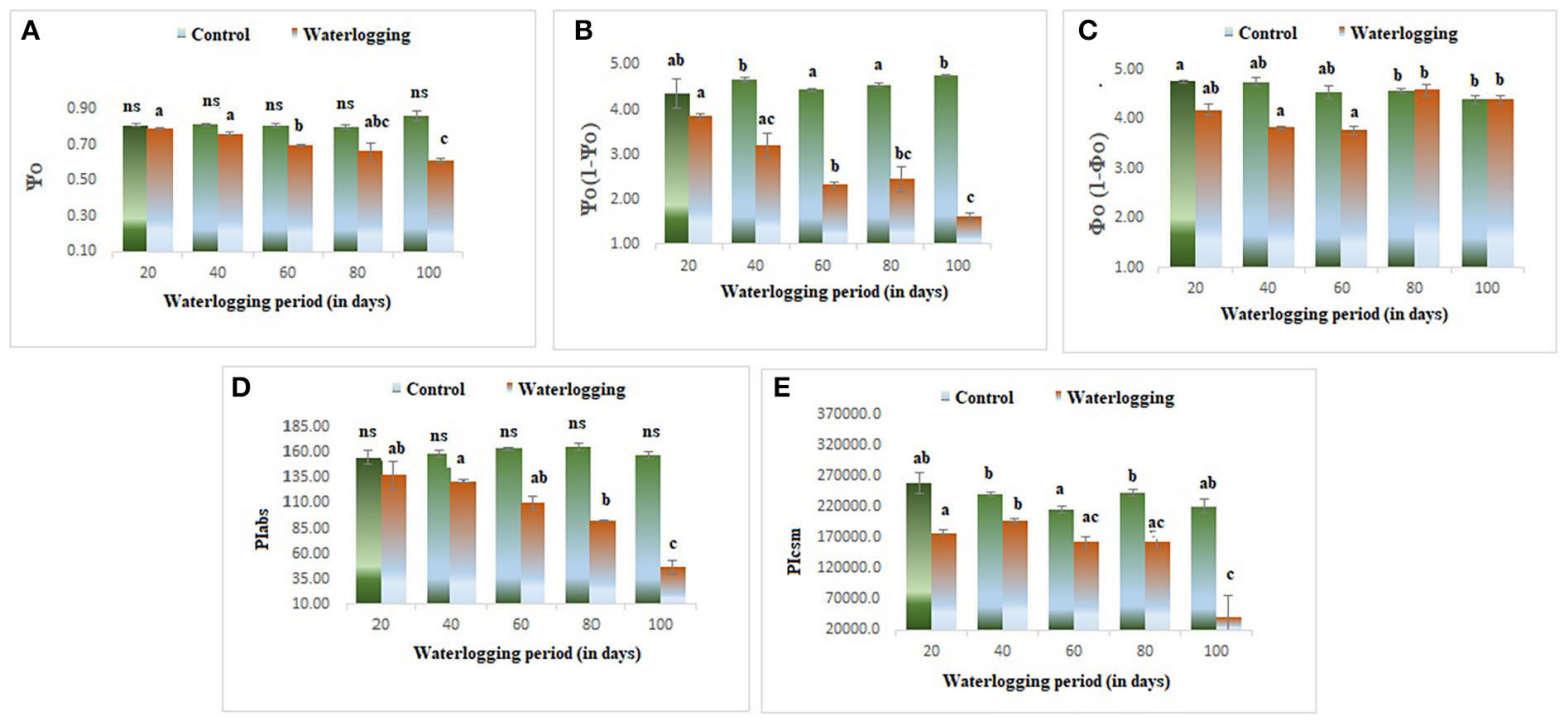
Waterlogging induced changes in **(A)** Ψ_o_ **(B)** Ψo (1 – Ψo) **(C)** φo (1 – φo) **(D)** PIabs **(E)** PIcsm.

The values of φP_o_ and φE_o_ declined in all waterlogging treated plants as compared to the control. However, the higher value of φD_o_ was reported in 80 and 100 days of waterlogged plants (Figure 8). Ψ_O_ and Ψ_O_/ (1 – Ψ_O_) were reduced in all treated plants as compared to the control in our study indicating a lowering in plant performance. Whereas φo/(1 – φ_o_) was decreased in 20, 40, and 60 days of waterlogged plants than control. Further, in 80 days and 100 days of waterlogged plants, the values of φ_o_/(1 – φ_o_) have remained the same as in control (Figure 9). Other performance indices such as PIabs and PIcsm were also lowered as compared to control in all waterlogging treated plants (Figure 9).

#### G-mean clustering and principal component analyses

**Figure 10:**
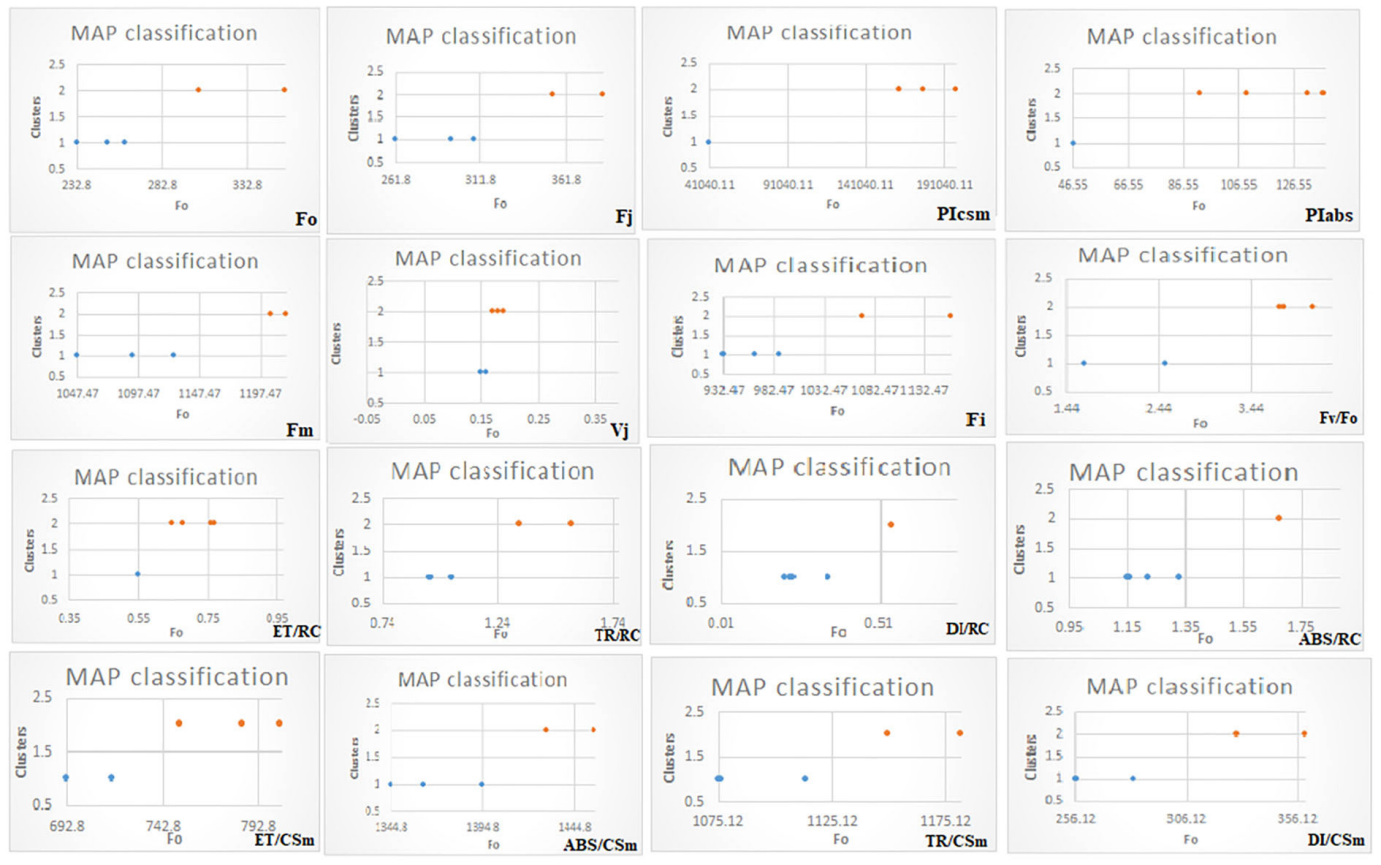
MAP classification model of G-mean clustering of different chlorophyll fluorescence parameters.

**Figure 11:**
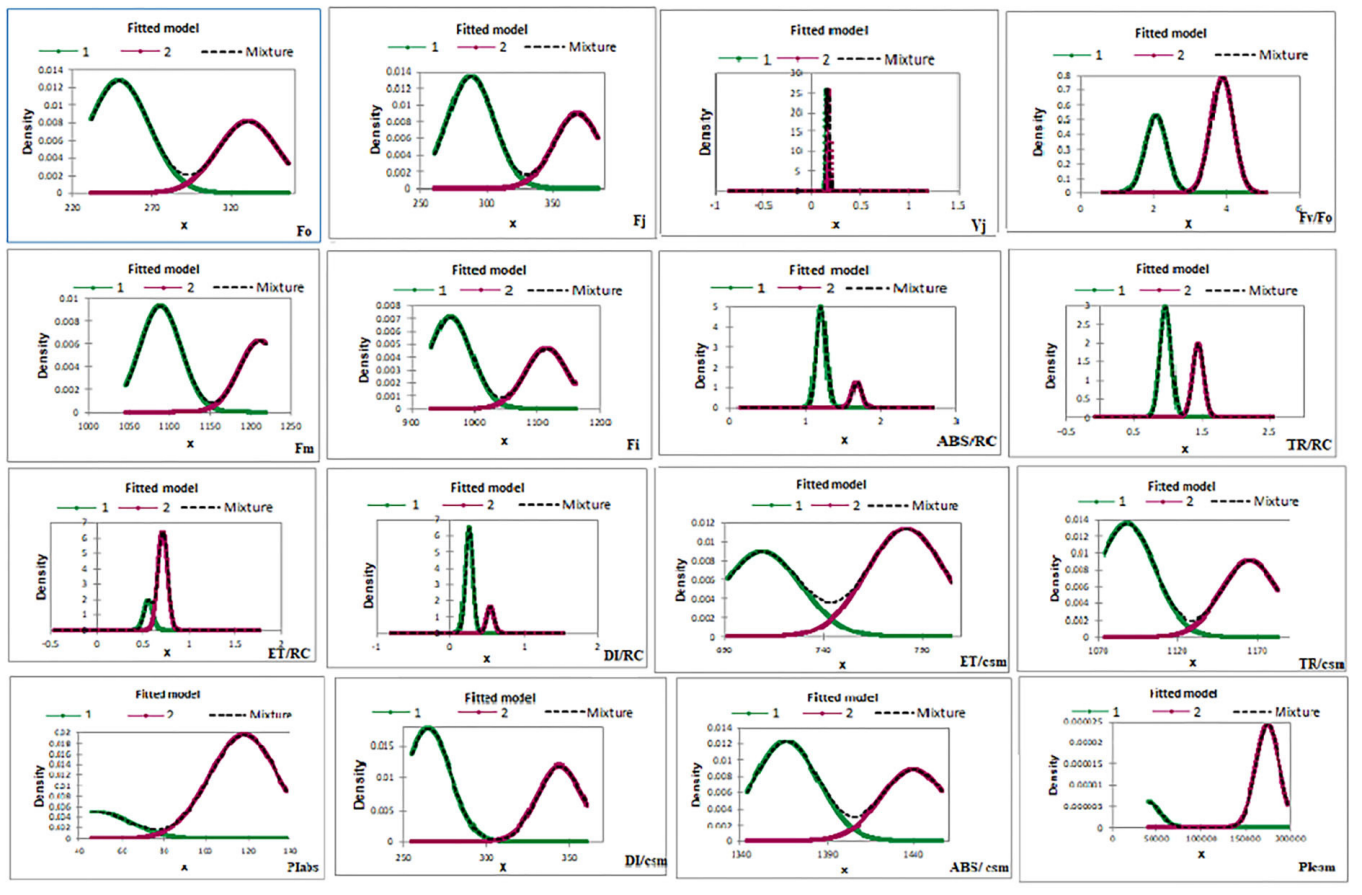
Energy-fitted model of G-mean clustering of different chlorophyll fluorescence parameters.

**Figure 12:**
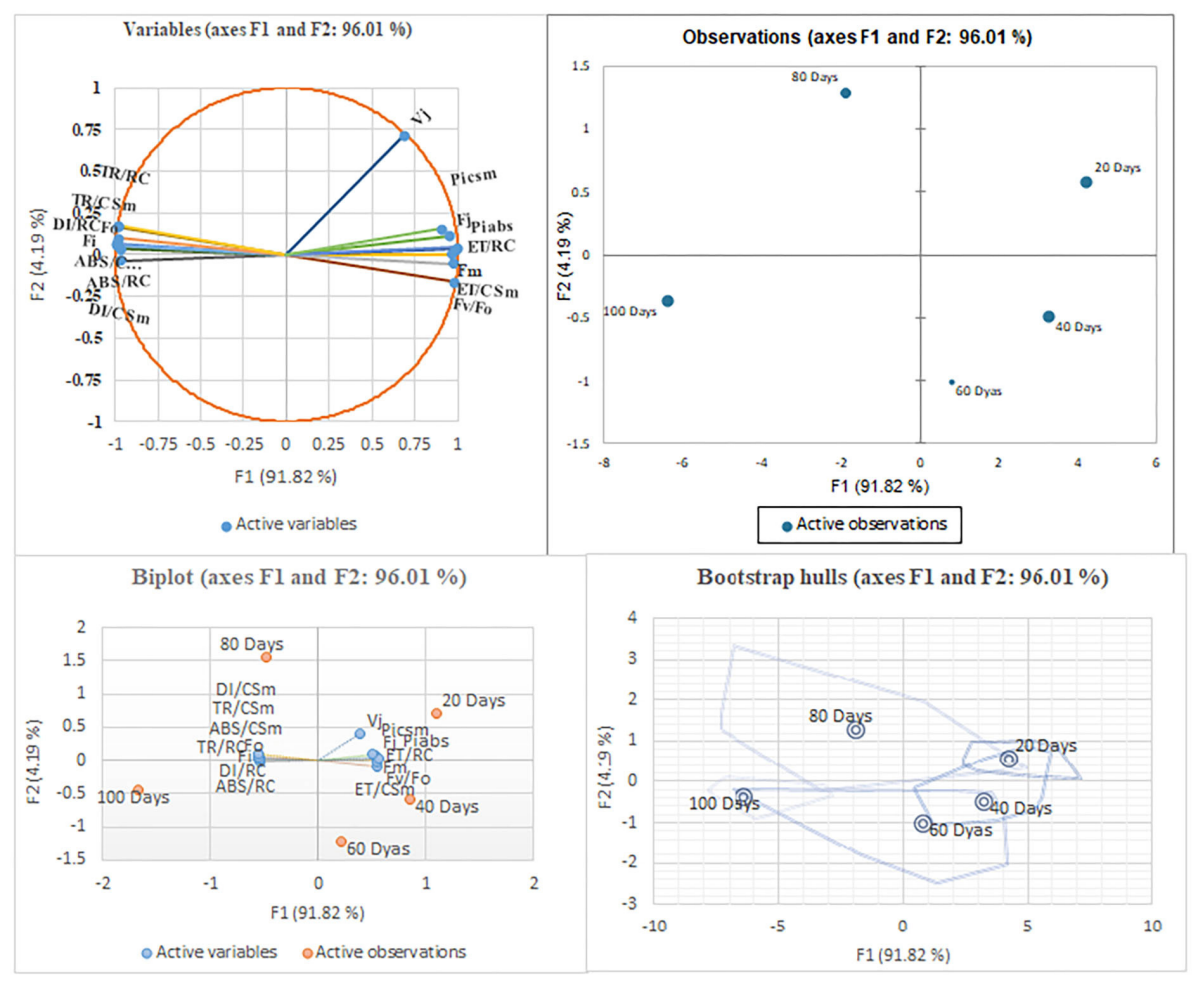
The principal component analysis with different waterlogging treatment conditions. The PCA is based on the chlorophyll fluorescence data. Arrows represent the chlorophyll fluorescence parameter on the corresponding dimensions (PC 1 and PC2), where PC 2 expressed most of the variability in the data.

**Figure 13:**
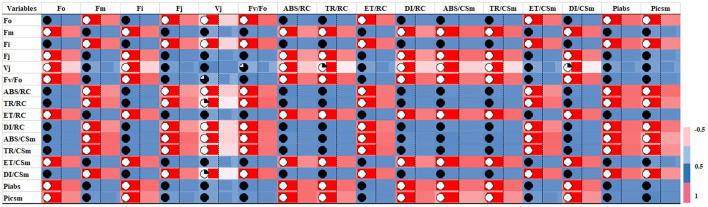
Heat map of correlation of chlorophyll fluorescence parameters showing the interrelationship between chlorophyll fluorescence parameters.

In G-mean clustering, the physiological parameters Fo, Fi, TR/RC, ABS/CSm, TR/CSm, and DI/CSm were affected similarly under waterlogging conditions in peanuts. ABS/RC and DI/RC were kept in the same group because they were affected more severely on 100 days of waterlogging than on 20, 40, 60, and 80 days of waterlogging. Other groups were (1) Fm and F_I_, (2) Fv/Fo, (3) ET/CSm, and (4) PIabs, PIcsm, and ET/RC. These groups contain parameters that were affected similarly under waterlogging treatment (Figures 10, 11). The PCA describes the correlation between different physiological parameters and the waterlogging duration (Figure 12). The results indicate that the fluorescence parameters were affected similarly in 40 and 60 days of waterlogging and that there was a 96.01% variation found in the studied physiological parameters. The heat map of the correlation between different physiological parameters can be seen in Figure 13. Black dots show the positive correlation between the parameters under waterlogging and white dots represents the negative correlation under waterlogging. Data indicates that Fo, Fi, ABS/RC, TR/RC, DI/RC, ABS/CSm, TR/CSm, and DI/CSm are positively correlated to each other whereas Fm, Fj, Vj, Fv/Fo, ET/RC, ET/CSm, Piabs, and Picsm are positively correlated to each other.

## Discussion

Peanuts are essential raw materials for food, pharmaceutical, and other sectors due to their high protein and oil content (Zeng et al., 2022). Furthermore, peanuts are important to the growth and economy of agriculture (Latif et al., 2013). Previous studies have suggested that waterlogging led to the rotting of peanut pods, which in turn resulted in a loss of production (Bishnoi and Krishnamoorthy, 1992; Zeng et al., 2021). Increasing greenhouse gas concentrations may have caused a twofold increase in the occurrence of extreme precipitation (Smethurst and Shabala, 2003). Therefore, a detailed evaluation system must be established for cultivation and breeding to determine the peanut's waterlogging tolerance.

Waterlogging stress damaged the cells and membrane systems and deteriorated the leaves' ability to photosynthesize (Smethurst and Shabala, 2003; Irving et al., 2007; Zhang X. et al., 2015; Singh et al., 2019). Waterlogged plants are susceptible to cellular damage and may develop irreversible metabolic dysfunctions that result in cell death (Pereira et al., 2015). Soil drainage that reduced waterlogging stress actually raised oxidative stress and possibly accelerated plant damage instead of enhancing plant performance (Hossain et al., 2009).

A study by Liu et al. (2014) showed that plants may reduce the amount of photosynthetic products they consume by slowing leaf growth and reducing the number of blades to adapt to the anoxic environment imposed by waterlogging stress. During the waterlogging, the control group continued to grow healthily, and after 20–40 days, just a few leaves started to show leaf curling and slight wilting. The morphological appearance of the peanut plants was unaltered under control conditions, though soggy plants began to mildly droop and shed after 20 days. The wilting, yellowing, and leaf-shedding intensified with prolonged waterlogging.

To evaluate how well-adapted and tolerant plants are to changes in their growth environment, several chlorophyll fluorescence properties can be assessed (Kuai et al., 2015). Research suggests that plants under non-stress conditions have a more functional photosynthetic reaction center than plants under stress. The physiological signal modifies in several ways whenever plants are subjected to waterlogging.

For example, when China wingnut (*Pterocarya stenoptera*) and Cork oak (*Quercus variabilis*) were subjected to waterlogging, a notable decline in maximal quantum efficiency (Fv/Fm) was seen (Yi et al., 2006). The maximum quantum yield of PS II photochemistry (Fv/Fm) was also lowered in field beans subjected to various days of waterlogging (Pociecha et al., 2008). The PSII photochemistry of *Medicago sativa* was similarly influenced by waterlogging. The reduction in Fv/Fm demonstrated the susceptibility of the photosynthetic apparatus to waterlogging and the plants' inability to regenerate rubisco under adverse conditions (Smethurst et al., 2005).

The fluorescence at 50 s (open) shows the quantity of Q_A_, the only primary accepter of quinone that has been oxidized. Moreover, it is interpreted as a symptom of irreparable photosystem II damage. Increased Fo is a marker of restricted LHC II dissociation and electron transport in all waterlogged treated plants (Kumar et al., 2020). However, the highest value of Fo, discovered in the DH-86 after 100 days of waterlogging, indicates permanent damage to the photosystem II as a result of a longer waterlogging period. All treated plants' lowered Fm reveals that the D1 protein's altered conformational shape changed the PSII electron acceptors' characteristics (Kumar et al., 2020; Singh et al., 2021; Bhatt et al., 2022). All photosynthetically active leaves may have lower PS II efficiency due to the lower value of Fm.

The chlorophyll fluorescence intensity at the J and I phase of the ChlF kinetics is denoted by the letters Fj and Fi, respectively. These time points depend on the kinetics of the photochemical reaction, which means that they may occur at different times depending on the physiological state of the plant and the settings of the experiment (levels). On the ChlF induction curve, J and I are normally considered the first and second inflection points or intermediary peaks, respectively. The physiological status of certain plants, algae, and cyanobacteria may be impacted by the abiotic stress (Clark et al., 2000; Stirbet, 2011; Grieco et al., 2015; Sunil et al., 2020) and thus the times of occurrence of these transitions will change.

The difference in Fv/Fo indicates that PS II's absorption of light energy was used to lower Q_A_'s efficiency and maybe cause vitality changes in PS II (Rao et al., 2021). It may demonstrate how plants are resistant to adverse environments (Zhang, 1999). When determining the maximum quantum yield of PSII, the parameter Fv/Fo, which accounts for simultaneous changes in Fm and Fo, is higher in the plant under waterlogging conditions than in plants retained as controls. The most delicate link in the photosynthetic electron transport chain is Fv/Fo (Mohammed et al., 2003). Either a reduction in Fv or an increase in Fo perhaps accounts for the decline in Fv/Fo. In the present study, Fo was reduced, leading to an increase in Fv/Fo (Nedbal et al., 2000). The reduced number and size of RC, which have also been reported in various plants exposed to disease and environmental stresses, is indicated by the lower value of Fv/Fo in treated leaves. This change in the rate of electron transport from PSII to the primary electron acceptors has also been reported in different plants exposed to disease and environmental stresses (Martinazzo et al., 2012; Janka et al., 2013). In plants under waterlogging, relative variable fluorescence at the J-step (2 ms) decreased. The PSII's primary quinine electron acceptor [Q_A_-/Q_A_ (total)] fraction was measured by Vj (Strasserf et al., 1995). The findings of our investigation show that the peanut plants' electron transport at the donor side of PSII was inhibited by waterlogging.

In waterlogged plants, specific energy fluxes such as ABS/RC and TR/RC increased. The ABS/RC ratio is calculated by dividing the total photons absorbed by Chl molecules across all RCs by the total number of active RCs. This is an excellent indicator of the average functional antenna size (Tsimilli-Michael et al., 2000). The ratio of active to inactive RCs affects it, and as the number of active centers fell, the ratio of ABS/RC rose. Interestingly, a lower ratio of chlorophyll a to b led researchers to believe that the PSII antennae were larger (Lichtenthaler et al., 1982).

Despite RC being less active, it is more effective at reducing plastoquinone as shown by the higher value of specific energy flux (ABS /RC, TR/RC). Because there are fewer active RC and more Q_A_ reduction, the reoxidation of reduced Q_A_ *via* electron transport in an active RC is diminished, as indicated by the decline in ET/RC. The ratio of the total untrapped excitation energy dissipation from all RCs to the number of active RCs is known as DI/RC. Heat, fluorescence, and energy transfer to other systems cause dissipation. The ratios of active/inactive RCs also have an impact. The electron overloading of the PSII driven by the observed increase in antenna size under waterlogging conditions would change a certain number of active RCs into dissipative ones. By converting violaxanthin to zeaxanthin under the influence of a strong proton gradient across the thylakoid membrane (pH), these dissipative reaction centers are able to release the majority of the energy as heat (Buschmann, 1999). The increase in excitation energy dissipation (DI/RC) in the waterlogging condition compared to the control was accompanied by a rise in ABS/RC values. In all plants that had been treated for waterlogging, the phenomenological energy fluxes ABS/CSm and TR/CSm dramatically increased.

Further, the quantum yields of primary photochemistry (φPo), electron transport (φEo), and dissipation (φDo) provide relevant evidence on the activity of electron transport at the PSII acceptor sites (Strasser et al., 2004). The decline in their values reflects the reduced electron transport from the waterlogged PSII acceptor site. Performance index (PIabs) is based on energy absorption and has a lower value in plants that are flooded. The RC's total activity increases as PIabs increase due to the RC's enhanced activity. The PI creates a single multi-parametric expression from the density of RCs in the chlorophyll bed (RC/ABS), excitation energy trapping (φPo), and conversion of excitation energy to electron transport (Ψo), which are three separate functional phases of photosynthesis (Tsimilli-Michael et al., 2000; Strasser et al., 2004). More effectively than Q_A_- is the capability of a trapped exciton to transport an electron into the electron transport chain. Because there were more active RCs and total energy usage efficiency was higher compared to the control, the PI decreased on all days when there was waterlogging.

## Conclusion

According to our findings, waterlogging conditions resulted in a decrease in plant metabolic activity. Depending on the species and duration of waterlogging period, plants respond differently to waterlogging. To better recognize the different reactions of the same variety under various waterlogging durations, the effects of various waterlogging durations on the peanut DH-86 variety were evaluated in the current study. The growth rate, photosynthetic pigments, antioxidative enzyme activity, and chlorophyll fluorescence parameters of the peanut were all significantly impacted by the 100-day waterlogging, which reduced the starch and soluble sugar content (photo assimilates). Overall, our study of chlorophyll fluorescence revealed that chlorophyll biosynthesis, electron transport system, photosystem II, and performance indices are the most sensitive parameters to different waterlogging conditions in *A. hypogaea*.

Because of the conformational alterations in PSII, a portion of the active reaction centers were modified to operate as dissipative ones, which lowered the photosynthetic efficiency of peanut DH-86 plants. Additionally, it was discovered that lowering the TR/DI parameter resulted in a decline in the overall photosynthetic performance (PI_abs_) of waterlogged plants. Understanding how key crops tolerate waterlogging can also aid in the development of increased nutritional values in crops.

## Data availability statement

The raw data supporting the conclusions of this article will be made available by the authors, without undue reservation.

## Author contributions

VS, SS, UB, and JS design the methodology. SS, UB, and JS perform the experiments and wrote the manuscript. AD, JM, and VS supervised the experiments and corrected the manuscript. All authors contributed to the article and approved the submitted version.

## Conflict of interest

The authors declare that the research was conducted in the absence of any commercial or financial relationships that could be construed as a potential conflict of interest.

## Publisher's note

All claims expressed in this article are solely those of the authors and do not necessarily represent those of their affiliated organizations, or those of the publisher, the editors and the reviewers. Any product that may be evaluated in this article, or claim that may be made by its manufacturer, is not guaranteed or endorsed by the publisher.
